# Studies of rhizobial competitiveness for nodulation in soybean using a non-destructive split-root system

**DOI:** 10.3934/microbiol.2017.2.323

**Published:** 2017-05-05

**Authors:** Ángeles Hidalgo, Francisco-Javier López-Baena, José-Enrique Ruiz-Sainz, José-María Vinardell

**Affiliations:** Department of Microbiology, Faculty of Biology, University of Seville, Avda, Reina Mercedes 6, 41012-Sevilla, Spain

**Keywords:** split-root system, competitiveness, nodulation blocking, soybean, *Sinorhizobium fredii*, *Bradyrhizobium japonicum*

## Abstract

Split-root systems (SRS) constitute an appropriate methodology for studying the relevance of both local and systemic mechanisms that participate in the control of rhizobia-legume symbioses. In fact, this kind of approach allowed to demonstrate the autoregulation of nodulation (AON) systemic response in soybean in the 1980s. In SRS, the plant main root is cut and two lateral roots that emerge from the seedlings after root-tip removal are confined into separate compartments. After several days of growth, these plants have two separate roots that can be inoculated with the same or with different bacteria, at the same or at different times. In this work, we have used a non-destructive SRS to study two different competitiveness relations between rhizobial strains in soybean roots. One of them is the competition for nodulation between two soybean-nodulating rhizobia: the slow-grower *Bradyrhizobium japonicum* USDA110 and the fast-grower *Sinorhizobium fredii* HH103. The second is the competitive blocking of *S. fredii* 257DH4 nodulation in the American soybean Osumi by *Sinorhizobium fredii* USDA257, which is unable to nodulate American soybeans. Our results showed that the competitiveness relationships studied in this work are mitigated or even avoided when the competitive strains are spatially separated in different compartments containing half-roots from the same plant, suggesting that competitive relations are more related to local plant responses. In our opinion, split-root systems are an appropriate approach to further study competitive relations among rhizobial strains.

## 1. Introduction

Rhizobia are soil proteobacteria that establish specific symbiotic relationships with leguminous plants resulting in the formation of nitrogen-fixing nodules in plant roots [1]. The nodulation process is a complex event that requires a molecular dialogue between the partners. In response to flavonoids exuded by the roots, rhizobia secrete specific signal molecules called Nod factors or LCOs (for lipochitooligosaccharides) that, after their perception by plant receptor-kinases, promote root hair curling and induce the development of root nodules in the plant [2],[3]. In most cases, rhizobia penetrate into the root moving along tubular structures formed by invagination of the root hair cell membrane. These tubular structures, called infection threads, reach the nodule symbiotic cells in which bacteria are released and differentiate into nitrogen-fixing bacteroids. In addition to Nod factors, other bacterial molecules, surface polysaccharides and proteins secreted by bacterial secretion systems, also play important roles in the establishment of an efficient symbiosis with the host plant [4].

Nitrogen is a limiting element for plant growth in most ecosystems [5],[6]. Legumes are the second most important plant family, only after *Graminiae*, in agronomical importance and offer the advantage of requiring low or null supply of nitrogen fertilizers when nodulated by either natural or inoculated population of effective rhizobia [7],[8]. In fact, the advantages of inoculating legume crops with high quality rhizobial inoculants over the use of chemical N-fertilizers are numerous and include economical as well as environmental benefits [9]. However, rhizobial inoculants often lack efficacy because of the competition problem, a phenomenon in which inoculated rhizobia fail to nodulate legumes because indigenous rhizobia populations are better adapted for root infection and, consequently, for nodule occupancy [10],[11],[12]. In the practice, the degree of settlement and persistence of an inoculant strain generally decreases when increasing densities of native rhizobial populations. For this reason, in order to be used as inoculants for legumes, rhizobial strains should be not only highly efficient in nitrogen fixation but also highly competitive for nodulation [8].

Soybean is the most important legume crop worldwide [8],[13]. This plant can be nodulated by different species of *Bradyrhizobium*, *Rhizobium*, *Mesorhizobium* and *Sinorhizobium* [14], including slow-growing (such as *Bradyrhizobium* spp.) and fast-growing rhizobia (such as *S. fredii* strains). In most cases, commercial soybean inoculants are elaborated with *Bradyrhizobium japonicum* (currently renamed as *B. diazoefficiens*) strains, such as USDA110, due to their high efficiency in fixing nitrogen [8],[15]. However, depending on both soil conditions (such as pH) and on soybean cultivar, *S. fredii* strains can have an advantage over *B. japonicum* for nodulating soybean [8],[16],[17].

*S. fredii* strains differ in their ability to nodulate different soybean (*Glycine max*) cultivars [4]. Thus, some strains, such as USDA257, can only nodulate Asiatic soybean varieties, whereas other strains, such as HH103, in addition, can also induce the formation of nitrogen-fixing nodules in American, commercially improved, soybean varieties. Indeed, one of the best studied *S. fredii* strains, NGR234, is not able to nodulate soybean [18]. The inability of strain USDA257 to nodulate American soybeans is caused by the secretion of effectors through a type 3 secretion system (T3SS). It has been suggested that the recognition of an effector by a specific plant receptor could trigger a defence response that could block nodulation [19],[20]. Thus, an USDA257 mutant, called 257DH4, unable to secrete proteins through this T3SS, induces nitrogen-fixing nodules in the soybean American variety McCall [21]. Curiously, the presence of the parental strain USDA257 blocks the ability of strain 257DH4 for nodulating this cultivar, which may suggest the induction of plant defence responses.

To our knowledge, competitiveness and nodulation-blocking relationships between rhizobial strains for nodulating legumes has been always studied by adding the competitor strains on the same root. In this work, we have used a split-root system for soybean in order to analyse whether these kind of relationships are due to local or to systemic effects.

## 2. Materials and Method

### 2.1. Microbiological techniques

*B. japonicum* USDA110 and *Sinorhizobium fredii* strains HH103 Str^R^, USDA257 Rif^R^ and 257DH4 were grown at 28 °C on yeast extract/mannitol (YM) medium [21]–[25]. When required, the media were supplemented with antibiotics (concentrations expressed in µg/mL): rifampicin (Rif), 50; streptomycin (Str), 400; neomycin (Neo), 50; tetracycline (Tet), 1.

### 2.2. Soybean split-root system in hydroponics conditions

**Figure 1.**
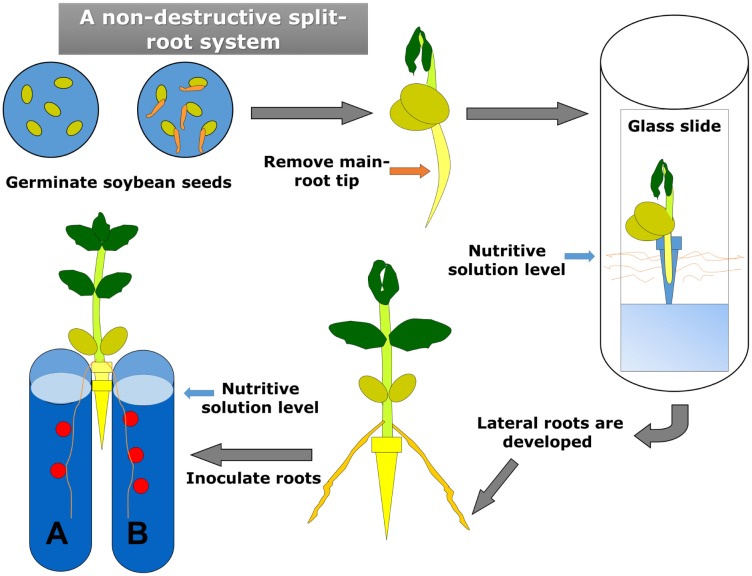
Schematic diagram of our non-destructive split-root system for soybean. Inoculated roots are located between a filter paper sheet and the glass wall of the tube, so they are visible along the complete experiment, allowing continuous scoring of the appearance of nodules without removing plants from the experiment.

In this work we have used a split-root system (SRS) for soybean which, unlike most SRS [26], operates in hydroponics conditions (Figure 1) [27]. Our method is non-destructive and allows continuous monitorization of nodule development in the same roots, as well as the possibility of sampling in a time-course manner. Briefly, *Glycine max* cv. Osumi seeds were surface sterilized and germinated. The root-tip of two days-old germinated seeds were excised and the manipulated seeds were incubated for 120 h in Fåhraeus nutritive solution in order to allow the development of lateral roots. Those seedlings with two lateral roots showing similar length and position were selected. After the excision of the remaining lateral roots, the selected seedlings were transferred to a twin-tube system containing Fåhraeus nutritive solution (pH 7.1) and each lateral root was placed in a tube. Each tube contained a sheet of filter paper, and the roots were placed between the filter paper and the tube wall, in such a way that the root remains extended and visible along the experiment. Every twin-tube system was protected from light by covering with a black plastic bag. When indicated, one or the two radical systems of each plant were inoculated with rhizobia. As a control, we have used single-root systems in which soybean plantlets were placed in single-tube systems containing a sheet of filter paper and Fåhraeus nutritive solution.

For *B. japonicum* USDA110 Tet^R^ Str^S^/*S. fredii* HH103 Tet^S^ Str^R^ competition for nodulation assays, the inoculum size for each root was 10^8^ bacteria. When coinoculated, the ratio was 1:1. Soybean plants were analysed 34 days after inoculation (dai). Nodule occupancy by *B. japonicum* USDA110 was determined by the presence of soybean nodule isolates able to grow on YMA supplemented with tetracycline (1 µg/mL). Isolates from nodules induced by *S. fredii* HH103 grew in YMA supplemented with streptomycin (400 µg/mL). Two independent experiments with 8 plants per treatment were carried out. These experiments gave similar results. Table 1 shows the results obtained in one of these experiments.

For *S. fredii* USDA257 Rif^R^/*S. fredii* 257DH4 Neo^R^ competition for nodulation assays, the inoculum size for each root was 3 × 10^7^ bacteria for USDA257 and 3 × 10^6^ bacteria for 257DH4. Soybean plants were analysed 41 dai. Nodule occupancy by 257DH4 was determined by growing the isolates on YMA supplemented with neomycin (50 µg/mL), the antibiotic-resistance marker of transposon Tn*5* (which was used to generate the mutation carried by this strain). No isolates corresponding to USDA257 (Rif, 50 µg/mL) were found from nodules formed in roots inoculated with both strains. Two independent experiments (giving similar results) with 8 plants per treatment were carried out. Table 2 shows the representative results obtained in one of these experiments.

### 2.3. Statistical analyses

When indicated, different treatments were pair-wise compared by using the Mann-Whitney non-parametrical test, α = 5%. When more than two treatments were compared, the One-Way ANOVA test (α = 5%) was employed.

## 3. Results and Discussion

### 3.1. Competition for nodulation assays between *Bradyrhizobium japonicum* USDA110 and *Sinorhizobium fredii* HH103 in soybean cv. Osumi

When two or more rhizobial strains able to nodulate a specific legume coexist in the same environment, these strains compete for nodulating this plant. The competitiveness ability of each strain is reflected in the percentage of nodules occupied by them. As previously mentioned, soybean plants can be mainly nodulated by both slow- (belonging to *Bradyrhizobium*) and fast-growing rhizobial (belonging to *Sinorhizobium*) strains. Previous studies have shown that the outcome of competition for nodulation between fast- and slow growing strains can be significantly influenced by the soybean cultivar used and also by the pH at which plants are grown [16],[17]. In general, nodule occupancy by *S. fredii* is predominant in alkaline conditions whereas *B. japonicum* strains are more competitive in neutral and acidic conditions. In addition, earlier studies showed that in greenhouse conditions (carried out at neutral or slightly acidic conditions) *Bradyrhizobium* outcompeted *Sinorhizobium* strains for nodulating soybean [28],[29].

In this work we have analysed the competition for nodulation relationships between the slow-growing *B. japonicum* USDA110 and the fast-growing *S. fredii* HH103 strains for nodulating soybean Osumi in plant chamber conditions. By using a split-root system, we have compared the competitiveness of these strains when each half-root (hereafter called A- or B-root) was inoculated with one of the strains and when each half-root was inoculated simultaneously with both strains. As a control, we have also used a single-root system in which the two strains were added at the same time on intact soybean roots. Results are shown in Table 1.

When both *B. japonicum* USDA110 and *S. fredii* HH103 were used for inoculating the same half-root, only the 0.5% of the nodules formed were occupied by HH103. A similar result was found in the single-root system, in which strain USDA110 was found as the only occupant of the 100% of the nodules formed. The number of nodules formed in single-roots (44.3 ± 10.72) was similar to the total number of nodules formed by A- and B-roots in the split-root system (48.75 ± 10.87). Although the initial pH of the plant nutritive solution was 7.1, along the experiment this value was progressively diminishing until acidic values of 4.1–4.3. Our results using the split-root system are in agreement with those obtained using intact roots [16],[17] since *B. japonicum* outcompeted *S. fredii* in both plant systems when plants were grown at neutral-acidic conditions.

When *B. japonicum* USDA110 and *S. fredii* HH103 were used to simultaneously inoculate different half-roots of the same plant in the split-root system, the output of nodule occupancy was different to that obtained when both strains were added to the same root (Table 1). Nodules appeared in both half-roots, although the half-roots inoculated with USDA110 formed a significantly higher number of nodules (B-roots, 31.14 ± 11.76) than those inoculated with HH103 (A-roots, 15.29 ± 5.22). Moreover, the fresh mass of nodules induced by USDA110 was significantly higher than that of those formed in the half-roots treated with HH103 (fresh weight of 323 ± 90 and 65 ± 40 mg/root, respectively), which indicates that the contribution of USDA110 to the development of the plant was considerably higher than that of HH103. The total number of nodules formed in the A- and B-roots (46.43 ± 11.56) was not significantly different to those observed when both strains were applied on the same root regardless the system (split-root or single-root) used. Our results indicate that the ability of USDA110 to completely outcompete HH103 when they are co-inoculated on the same root is somehow weakened when both strains are spatially separated and do not compete for the same infection places.

**Table 1.** Competition experiments between *Sinorhizobium fredii* HH103 (HH) and *Bradyrhizobium japonicum* USDA110 (USDA) in “Single-root” (SR) and in the “Split-root system” (SRS). Data were taken at 34 dai.

		**Root**		
	**Treatment**	**Root A**	**Root B**	**Roots A + B**

**Number of nodules**	**SRS: HH/USDA**	15.29 ± 5.22*	31.14 ± 11.76*	46.43 ± 11.56**^A^**
	**SRS: HH + USDA/HH + USDA^a^**	22.63 ± 8.47	26.13 ± 6.94	48.75 ± 10.87**^A^**
	**SR: HH + USDA^b^**	N/A	N/A	44.3 ± 10.72**^A^**
**Nodule fresh-weight (mg)**	**SRS: HH/USDA**	65 ± 40*	323 ± 90*	388 ± 104**^A^**
	**SRS: HH + USDA/HH + USDA**	231 ± 53	258 ± 42	489 ± 53**^A^**
	**SR: HH + USDA**	N/A	N/A	449 ± 36**^A^**
**Final pH**	**SRS: HH/USDA**	4.93 ± 0.85	5.21 ± 0.51	N/A
	**SRS: HH + USDA/HH + USDA**	4.11 ± 0.17	4.34 ± 0.42	N/A
	**SR: HH + USDA**	N/A	N/A	4.2 ± 0.9

	**Treatment**	**Plant-top dry-weight (g)**		

	**SRS: HH/USDA**	0.75 ± 0.22**^A^**		
	**SRS: HH + USDA/HH + USDA**	0.97 ± 0.15**^A^**		
	**SR: HH + USDA**	0.96 ± 0.07**^A^**		

Data shown are the mean ± standard deviation of the mean and represent averages of 8 plants. Determinations were made 34 days after inoculation.

SRS, split-root system; SR, single root; N/A, non-applicable.

**^a^** 99.5% of the nodules were occupied by USDA110.

**^b^** 100% of the nodules were occupied by USDA110.

For “Number of nodules” and “Nodule fresh weight”, data in the same row marked with an asterisk are significantly different (Mann-Whitney non-parametrical test, α = 5%.).

For “Number of nodules”, “Nodule fresh weight”, and “Plant-top dry-weight (g)”, data in the same column with the same letter were not significantly different (One-Way ANOVA, α = 5%.).

**Figure 2.**
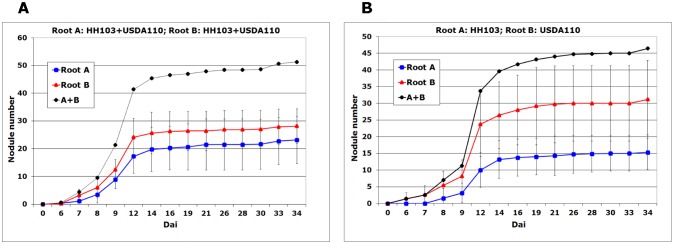
Kinetics of nodulation of soybean in split-root system. In both cases, half-roots A and B were simultaneously inoculated. Panel A, root A: *S. fredii* HH103 + *B. japonicum* USDA110, root B: *S. fredii* HH103 + *B. japonicum* USDA110. Panel B, root A: *S. fredii* HH103, root B: *B. japonicum* USDA110.

Figure 2 shows the kinetics of nodulation of the A- and B-roots in the split-root system. When each half-root was inoculated with both strains (Figure 2A), nodules were scored in both half-roots at 6 dai. In contrast, when the B-root was inoculated with USDA110 and the A-root with HH103, nodules appeared at 6 and 8 dai respectively (Figure 2B). These results indicate that the higher competitiveness ability of USDA110 when compared to HH103 may be due to the fact that the slow-grower USDA110 is able to infect soybean roots faster than the fast-grower HH103. This difference in the speed of infection appears to be crucial when USDA110 and HH103 are applied on the same root, resulting in an almost complete out-competition of the latter strain. When both strains are used to inoculate separate half-roots, although HH103 infections are delayed with respect to those of USDA110, the former strain is still able to induce the formation of some nodules. However, since the plant controls the total number of nodules formed, and nodules start to develop earlier in the half-roots treated with USDA110, the number of nodules induced for this strain is higher than that of nodules occupied by HH103.

### 3.2. Studies about the ability of *Sinorhizobium fredii* USDA257 to block nodulation of USDA257 T3SS defective mutant (257DH4) in soybean Osumi

Some rhizobia, including *B. japonicum* and *S. fredii* strains, possess a T3SS that delivers effector proteins (called Nops for Nodulation outer proteins) into host cells in order to manipulate host signalling and suppress plant defence responses [4],[30]. The role of Nops in symbiosis is highly dependent on the specific symbiotic pair (rhizobial strain/legume) investigated. Thus, the absence of Nops can be beneficial, neutral, or detrimental for any particular interaction. In some particular cases plant recognition of bacterial Nops can even completely block nodulation. This has been proved to be the case in the interaction of *S. fredii* USDA257 with American soybean cultivars, such as Mc Call or Williams 82 [19],[21]. In fact, a USDA257 mutant derivative unable to deliver Nops, called 257DH4, is able to effectively nodulate Mc Call and other American soybean cultivars [21]. However, this 257DH4 nodulation capacity is sensitive to the presence of the parental strain USDA257 in the soybean rhizosphere, which indicates that secretion of Nops by USDA257 can block nodulation of American soybeans with mutant 257DH4.

Our non-destructive method for split-root system studies has been used to investigate whether the incapacity of 257DH4 to nodulate American soybean in the presence of USDA257 is due to a local effect or this blockage can be translocated from one lateral half-root to the other. For this purpose, several combinations of both strains were applied to A- and B-roots of the American soybean cultivar Osumi. As expected, when both half roots were inoculated with USDA257, plants were devoid of effective nodules at the end of the experiment. In contrast, plants in which A- and B-roots had been inoculated with 257DH4 formed a similar number of nodules (about 24), resulting in 48.8 ± 17.7 nodules/plant at 41 dai. Plants in which A- and B-roots had been inoculated with a mixed inoculum (USDA257 and 257DH4) showed a three-fold reduction in the number of nodules formed (about 15 nodules per plant that were equally divided in both lateral-roots). When USDA257 was applied to the A-root and 257DH4 to the B-root, nodules only developed in the B-root and the total number (26.3 ± 6.6) of nodules formed was similar to that scored for each A- and B-root (24.5 ± 13 and 24.3 ± 8.3) of plants in which both half-roots were only inoculated with 257DH4. These results indicate that the blocking effect of USDA257 over the nodulation capacity of 257DH4 only occurs when both strains are placed together on the same lateral root. This hypothesis was further confirmed when A-roots were inoculated with a mixture of USDA257 and 257DH4 and B-roots were only treated with 257DH4. In this case, the number of nodules formed on A- and B-roots was significantly different (6.7 ± 3.1 on A-root and 28.1 ± 8.5 on B-root), indicating that the blocking capacity of USDA257 over 257DH4 nodulation was restricted to those roots in which both strains are present.

Legume plants control the number of nodules formed in order to avoid excessive costs due to nodule formation and nitrogen fixation. This process, which is termed autoregulation of nodulation (AON), depends on a systemic mechanism controlled by a CLAVATA1-like receptor kinase [31]. First observations of this regulation were done by using a split-root system in soybeans [32]. These results showed a suppression of nodule development if inoculation of the B-root was delayed respect that of A-root. Symbiotically compatible rhizobia were used in this work. In our system, this phenomenon is clearly observed when inoculation of the B-root is delayed 6 days respect inoculation of the A-root. This reduction in nodule number is even higher when the inoculation of the B-root is delayed for 9 days [27]. In order to investigate whether the blocking activity of USDA257 over nodulation of 257DH4 with Osumi soybean is due to the onset of AON responses and/or to another phenomenon, two additional experiments were carried out (Table 2). On both assays, inoculation of the B-root with 257DH4 was delayed 9 days respect inoculation of the A-root. Mutant 257DH4 or its parental wild-type strain USDA257 were used to inoculate A-roots. As expected, inoculation of A-roots with the symbiotically-compatible 257DH4 inoculant provoked a reduction (three fold) of the number of nodules formed by B-roots (26.8 ± 11.4 in A-roots and 9.1 ± 4.5 in B-roots), and this decrease can be assigned to the AON mechanism. However, when A-roots were inoculated with the symbiotically incompatible USDA257, the number of nodules induced by 257DH4 on B-roots (21.1 ± 9.4) was not significantly different to that of B-roots inoculated with 257DH4 when A-roots were simultaneously inoculated with USDA257 (26.3 ± 6.6). These results indicate that the presence of USDA257 in one half-root does not affect nodulation of the other half-root by 257DH4, regardless inoculation of both half-roots was simultaneous or inoculation with 257DH4 was delayed.

**Table 2.** Use of the “split-root system” for studying the ability of *S. fredii* USDA257 for blocking soybean Osumi nodulation by the USDA257 T3SS defective mutant 257DH4.

**Inoculants^a^ (Root A/Root B)**	**Number of nodules (41 dai)**		
	**Root A**	**Root B**	**A + B**
**USDA257/USDA257**	0^A^	0^A^	0^A^
**DH4/DH4**	24.5 ± 13.0^B^	24.3 ± 8.3^C, D^	48.8 ± 17.7^D^
**USDA257/DH4**	0*^, A^	26.3 ± 6.6*^, C, D^	26.3 ± 6.6^C^
**USDA257 + DH4/DH4**	6.7 ± 3.1*^, A^	28.1 ± 8.5*^, D^	34.9 ± 9.5^C^
**USDA257 + DH4/USDA257 + DH4**	7.0 ± 3.6^A^	8.0 ± 3.5^B^	15.0 ± 4.2^B^
**DH4/DH4 (+9)^b^**	26.8 ± 11.4*^, B^	9.1 ± 4.5*^, B^	35.9 ± 14.7^C^
**USDA257/DH4 (+9)^b^**	0*^, A^	21.1 ± 9.4*^, C^	21.1 ± 9.4^B, C^

**^a^** 257DH4 is referred as DH4 in this table.

**^b^** Inoculation of root B was delayed 9 days with respect to that of root A.

Data shown are the mean ± standard deviation of the mean and represent averages of 8 plants.

For Root A and Root B: data in the same row marked with an asterisk are significantly different (Mann-Whitney non-parametrical test, α = 5%.).

For Root A, Root B and A + B: data in the same column with the same letter were not significantly different (One-Way ANOVA, α = 5%).

## 4. Conclusions

Rhizobial competition for nodulation could be defined as the capacity of a particular strain to occupy a percentage of nodules in competition with other rhizobia present in the rhizosphere before the AON induced by the legume blocks the formation of further nodules. Split-root systems allow investigations aimed at revealing the relevance of local and systemic mechanisms that participate in the control of rhizobia-legume symbioses, as well as in the determination of nodule occupancy by any particular rhizobial strain. Kosslak and Bohool [32] demonstrated the existence of AON systemic responses in inoculated soybean roots by using the split-root system. In this work, we have used a non-destructive split-root system to study competitive relations between rhizobial strains that are soybean symbionts. Our results show that this kind of interaction are mitigated or even avoided if the competitive strains are spatially separated in different compartments containing half-roots originated from the same plants. These results indicate that direct competition for colonization of root infectious foci could be important for determining nodule occupancy in a time course process that is ultimately governed by the AON response.
